# Sexual homomorphism in dioecious trees: extensive tests fail to detect sexual dimorphism in *Populus*

**DOI:** 10.1038/s41598-017-01893-z

**Published:** 2017-05-12

**Authors:** Athena D. McKown, Jaroslav Klápště, Robert D. Guy, Raju Y. Soolanayakanahally, Jonathan La Mantia, Ilga Porth, Oleksandr Skyba, Faride Unda, Carl J. Douglas, Yousry A. El-Kassaby, Richard C. Hamelin, Shawn D. Mansfield, Quentin C. B. Cronk

**Affiliations:** 1 Department of Forest and Conservation Sciences, Faculty of Forestry, University of British Columbia, Forest Sciences Centre, Vancouver, BC V6T 1Z4 Canada; 2 Department of Dendrology and Forest Tree Breeding, Faculty of Forestry and Wood Sciences, Czech University of Life Sciences, Prague, 165 21 Czech Republic; 3 Saskatoon Research and Development Centre, Agriculture and Agri-Food Canada, Saskatoon, SK S7N 0X2 Canada; 4 United States Department of Agriculture-Agricultural Research Service (USDA-ARS), Corn and Soybean Research, Wooster, OH 44691 USA; 5 Department of Wood Science, Faculty of Forestry, University of British Columbia, Forest Sciences Centre, Vancouver, BC V6T 1Z4 Canada; 6 Département des sciences du bois et de la forêt, Faculté de foresterie, de géographie et de géomatique, Université Laval, Québec, QC G1V 0A6 Canada; 7 Department of Botany, University of British Columbia, Vancouver, BC V6T 1Z4 Canada; 8 Present Address: Scion (New Zealand Forest Research Institute Ltd.), Whakarewarewa, Rotorua 3046 New Zealand

## Abstract

The evolution of sexual dimorphism and expansion of sex chromosomes are both driven through sexual conflict, arising from differing fitness optima between males and females. Here, we pair work in poplar (*Populus*) describing one of the smallest sex-determining regions known thus far in complex eukaryotes (~100 kbp) with comprehensive tests for sexual dimorphism using >1300 individuals from two *Populus* species and assessing 96 non-reproductive functional traits. Against expectation, we found sexual homomorphism (no non-reproductive trait differences between the sexes), suggesting that gender is functionally neutral with respect to non-reproductive features that affect plant survival and fitness. Combined with a small sex-determining region, we infer that sexual conflict may be effectively stymied or non-existent within these taxa. Both sexual homomorphism and the small sex-determining region occur against a background of strong environmental selection and local adaptation in *Populus*. This presents a powerful hypothesis for the evolution of dioecious species. Here, we suggest that environmental selection may be sufficient to suppress and stymy sexual conflict if it acts orthogonal to sexual selection, thereby placing limitations on the evolution of sexual dimorphism and genomic expansion of sex chromosomes.

## Introduction

The evolution of dioecy (separate male and female individuals) is primarily a mechanism to increase outcrossing. It is widespread in animals, but also includes numerous plants (~5–6% of angiosperm species and 43% of plant families)^1^. Similar to animal taxa, dioecious plant species investigated to date also show sexual dimorphism outside primary sexual characteristics (e.g., morphological, physiological, and/or life-history traits)^2–5^. Sexual dimorphism is presumed to result from sexual selection between genders, specifically through antagonistic selection driven by sexual conflict^4, 6–10^. This conflict arises from different fitness optima in males and females due to their distinctive reproductive strategies; trait values that are optimal in males may be suboptimal in females, and vice versa. Sexual conflict also has profound effects on the genomic architecture of the sex-determining region (SDR) and evolution of sex chromosomes in dioecious species^11–14^. Under sexual conflict, there is an advantage for the genes involved to translocate into a non-recombining SDR and alleles optimized for a particular sex can become stably associated with that sex without the advantageous combination being broken by recombination.

In theory, SDRs are expected to spread rapidly under sexual selection, eventually occupying the whole or major part of a chromosome, and ultimately leading from homomorphic to heteromorphic sex chromosomes. Yet, the recent discovery of the Y chromosome in cottonwoods and poplars (*Populus trichocarpa* Torr. & A. Gray and related species) illustrates that theoretical expectations are not always met in dioecious plants. In these species, the SDR has a genomic architecture that is relatively old (~7 Ma) but has remained very small and compact (up to 12 genes spanning ~100 kbp on chromosome 19)^15^. This is one of the smallest SDRs characterized in complex eukaryotes to date, even compared to other plant species with homomorphic sex chromosomes, such as strawberry (240 kbp) and papaya (9 Mbp)^14, 16–18^. One potential explanation for a small SDR may be a low level of sexual selection through reduced or suppressed sexual conflict over time^17^. Yet, low sexual selection might also reduce the occurrence of sexually dimorphic traits (outside primary sexual characteristics). While *P. trichocarpa* itself has never been examined, previous studies in *Populus* (aspen and poplar species) have indicated either strong (e.g., refs 19–29) or partial^30–32^ sexual dimorphism in both juvenile and adult plants, a result seemingly at odds with low sexual conflict. To date, only one study testing European aspen (*P. tremula* L.) failed to find significant sexual dimorphism considering leaf transcriptomic profiling and a limited number of morphological, biochemical and herbivory traits^33^. A full phenotypic study is therefore needed in order to resolve this apparent paradox.

In plants, selection for sexual dimorphism in secondary characteristics affecting fitness, such as growth, vigour, physiology, disease susceptibility and lifespan, may have strong ecological implications^4, 7^. Nevertheless, it is also possible that sex-based phenotypic variation may be much smaller compared to variation within a species reflecting adaptation to environmental heterogeneity^34–36^. Thus, investigations of sexual dimorphism in any species must be carefully evaluated against a background of strong adaptive population differentiation where unbalanced sampling might lead to spurious conclusions. In aspens, poplars, and cottonwoods, there is abundant and robust evidence for strong adaptation to heterogeneous environments^37–46^. Consequently, it is possible that the inconsistency between a small SDR in these taxa and reports of strong sexual dimorphism arises from differences between sexes being misreported. This might be due to: i) small sampling of individuals without consideration for heritable phenotypic variation relating to environment, ii) inadequate controls for population structure or relatedness between tested individuals, and iii) the unpopularity of reporting and publishing negative results.

In this study, we examined the extent of dimorphism in secondary sexual characteristics within *P. trichocarpa* (and related species, *P*. *balsamifera* L.) considering the strong patterns of environmental and local adaptation in these dioecious species. We used a genome-wide association study (GWAS) with an expanded population of *P. trichocarpa* to verify the extent of the SDR and sex-associated markers, following previous work identifying the Y chromosome^15^. Using results from the GWAS to identify sex-predictive SNPs, we assigned genders to 904 *P. trichocarpa* and 473 *P. balsamifera* genotypes, most of which were extensively phenotyped but had no observations of flowering. We investigated 96 non-reproductive functional and fitness-related traits (spanning phenology, physiology, growth and biomass accumulation, leaf and wood anatomy, survivorship, disease resistance, leaf biochemistry, and wood composition) in both sexes, correcting for bias from patterns that reflect demography and local adaptation. Our extensive tests for evidence of sex-based differences assessed the same genotypes throughout seasons, across years, occurring at different plantation sites, and under manipulated growth conditions, in the largest test for dimorphism in secondary sexual characteristics of any dioecious plant species to our knowledge.

## Results

### Sex-based GWAS and gender identifications using sex-predictive SNPs

**Figure 1:**
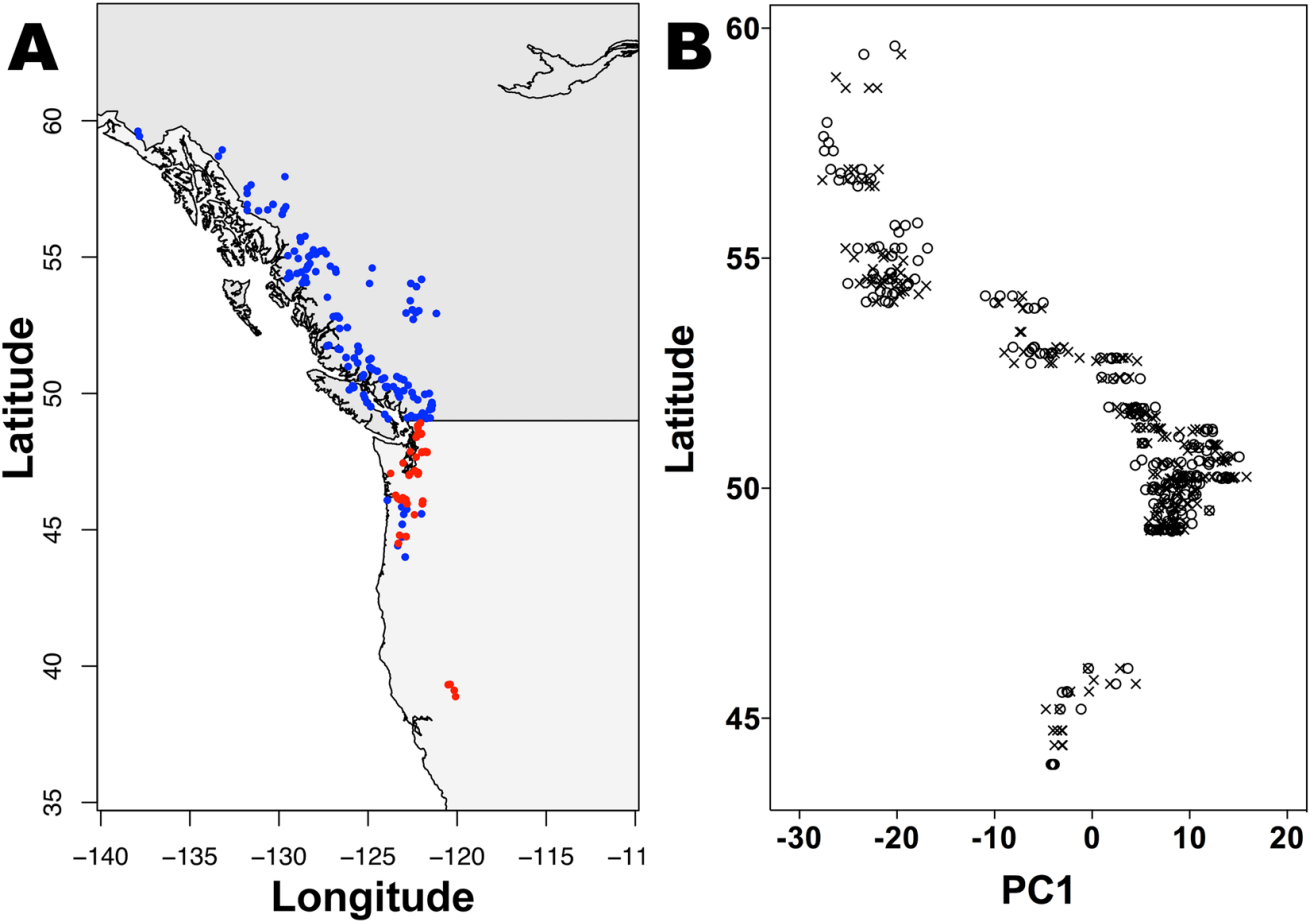
Distribution of *Populus trichocarpa* populations across western North America where individuals were sampled for genotypic and phenotypic data. (**A**) Physical localities of individuals collected throughout the species range. Blue dots represent 142 collection locations of 482 sex-identified genotypes used in this study obtained from the British Columbia Ministry of Forests, Lands & Natural Resource Operations (FLNRO). Red dots represent 353 collection locations of 448 sex-identified genotypes used in this study obtained from GreenWood Resources, Portland, OR and the BioEnergy Science Center, Department of Energy, Oak Ridge, TN (GW/BESC). Image generated using “maps” and “map data” packages in R v.3.3.1 (https://www.r-project.org/). (**B**) Latitudinal distribution of males and females from the FLNRO collection (ranging from 44.0–59.6 °N) using principal components analysis with 8 k non-sex associated SNPs. Sex is uncorrelated with main axis of population structure (PC1) which follows the species distributional geography (latitude). Circles represent male accessions and crosses represent female accessions. Image generated using Prism 7 (GraphPad Software, Inc.).

To verify the extent of the SDR and determine sex-predictive SNPs, we used GWAS with 126 flowering *P. trichocarpa* individuals (67 females, 59 males). Trees originated from a collection made by the British Columbia Ministry of Forests, Lands & Natural Resource Operations (FLNRO) across the northern two-thirds of the species’ natural range (44.0–59.6°N, 121.2–137.9°W), encompassing distantly related genotypes^43, 46^. All 126 genotypes were grown at Totem Field, University of British Columbia, BC (Fig. 1A, Table S1) and were fully sequenced^47^. This population was used to expand (with greater power) on previous work that revealed the sex locus in *P. trichocarpa* using 52 individuals of known sex^15^.

**Figure 2:**
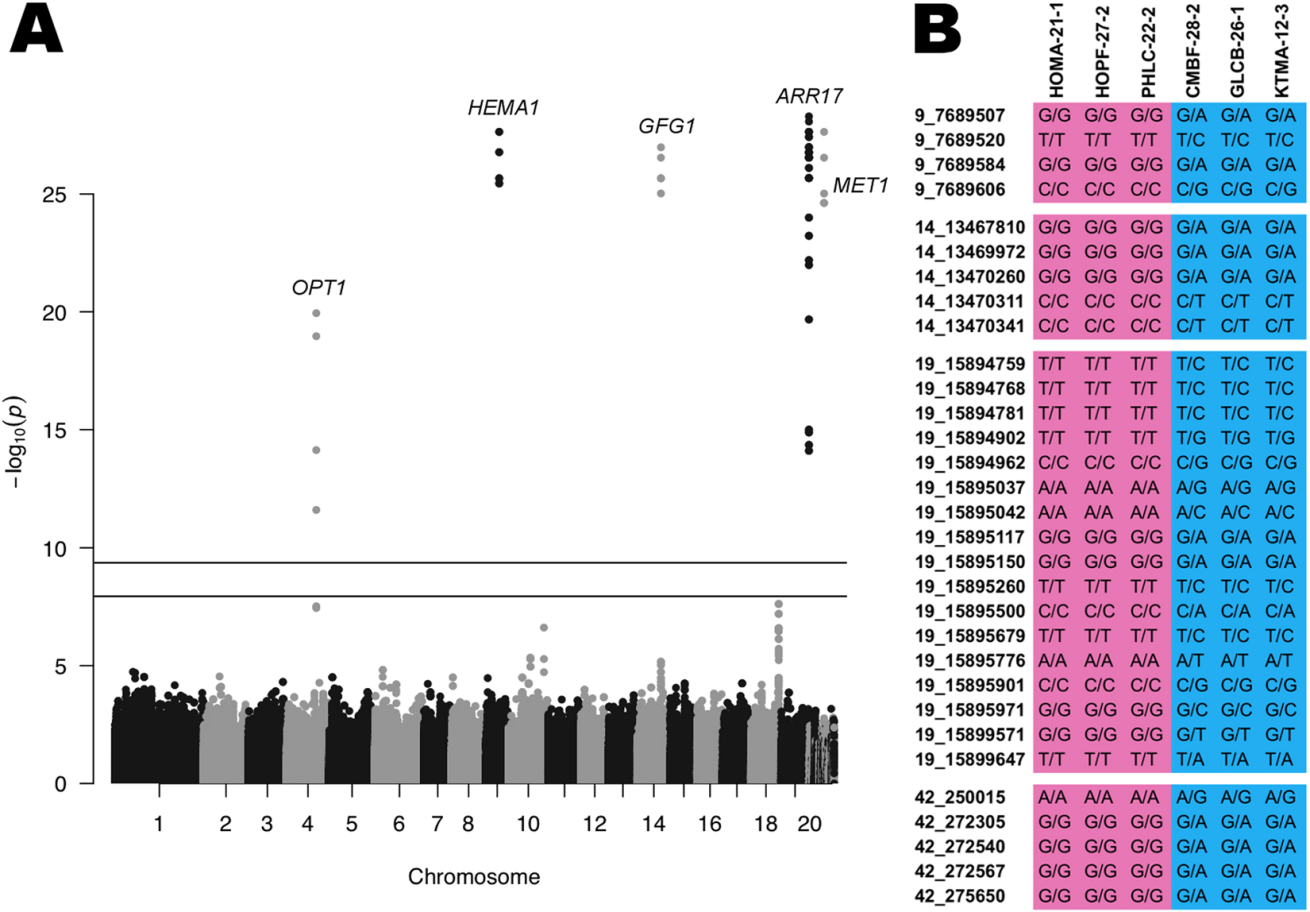
Suite of sex-specific SNPs in *Populus trichocarpa* identified by a genome-wide association study (GWAS). (**A**) Manhattan plot of 2.2 M SNPs from GWAS (−log_10_ *P*-values) assessing genders in 126 *P. trichocarpa* trees (67 females, 59 males). SNPs annotated to the v3.0 *P. trichocarpa* reference genome are mapped to Chr01–19 (including unassembled scaffolds) (full SNP results and *P*-values listed in Tables 1 and S3). The five genes uncovered by GWAS are annotated to different chromosomes in v3.0 of the reference genome due to genomic misassembly of the sex locus, as all previous studies indicate a single genetic region located on chromosome 19. The lower horizontal line indicates the −log_10_ *P*-value corresponding to a Bonferroni correction for multiple testing at α = 0.05 (significance at *P* < 2.3^e-8^) and the upper line at α = 0.001 (significance at *P* < 4.5^e-10^). (**B**) 32 strongly predictive, sex-specific SNPs in *P. trichocarpa* illustrating clear sex-identification in six representative genotypes previously unidentified by flowering or other methods. SNPs are listed across the top including chromosome and position (annotated to v3.0 of the *P. trichocarpa* genome) and coloring identifies allelic sex-specificity (pink = female, blue = male). Full results are listed in Table S2.

In our study, GWAS with 126 genotypes and 2.2 M filtered, bi-allelic SNPs (see Materials and Methods) uncovered 42 significant SNPs under the threshold for Bonferroni multiple testing correction at α = 0.05. Significant sex-associated SNPs occurred within only five genes annotated to the *P. trichocarpa* reference genome v3.0 (Fig. 2A, Table 1) and *P*-values for all SNPs (2.47 × 10^−12^ to 5.09 × 10^−29^) were considerably lower than our Bonferroni-adjusted cut-off (*P* < 2.26 × 10^−8^). These results were reduced from the number of SNPs and gene markers previously described^15^ and our analysis using a larger sample size indicated that there is no evidence for an extension to the SDR (beyond that of the previous study). Results were unchanged using either a model to correct for inherent population structure (Fig. 1B) or a simple logistic regression model (with no population structure implicated) (Fig. S1). While the five genes uncovered by GWAS were annotated to different chromosomes in v3.0 of the reference genome (Table 1), we consider that this results from genomic misassembly. The previous assessment underscored the complete genetic linkage of these markers, the evidence for a single genetic region, and other studies placing the SDR on chromosome 19 (see discussion in ref. 15).

**Table 1:** Genes and SNP markers uncovered by genome-wide association study (GWAS) for sex (male vs. female) in *Populus trichocarpa*.

Gene v3.0 (Gene v2.2, *Arabidopsis* homolog)	SNP (v3.0)	*P*-value (GWAS)^a^	Field flowering^b^	
			% Female (XX)	% Male (XY)
Potri.004G145400	16861660	1.14^e-20^	**99**	89
(POPTR_0004s15263, *OPT51*)	16861666^*^	1.08^e-19^	**99**	86
	16861972^†^	2.47^e-12^	72	90
	16862276^†^	7.39^e-15^	**100**	63
Potri.009G080600	7689507^*^	2.16^e-26^	**100**	**100**
(POPTR_0009s08410, *HEMA1*)	7689520^*^	3.57^e-26^	**100**	**100**
	7689584^*^	2.31^e-28^	**100**	**100**
	7689606	1.68^e-27^	**100**	**96**
Potri.014G168400	13467810	2.16^e-26^	**100**	**100**
(POPTR_0014s16640, *GFG1*)	13469972	1.05^e-27^	**100**	**100**
	13470260	9.50^e-26^	**100**	**99**
	13470311	2.87^e-27^	**100**	**100**
	13470341	2.16^e-26^	**100**	**99**
Potri.019G133600	15894544^†^	7.89^e-15^	83	**100**
(POPTR_0019s15410, *ARR17*)	15894759	3.82^e-28^	**100**	**100**
	15894768	1.73^e-27^	**100**	**100**
	15894781^*^	2.87^e-27^	**100**	**100**
	15894865^†^	1.00^e-15^	83	**99**
	15894902^*^	1.05^e-27^	**100**	**100**
	15894962^*^	5.09^e-29^	**100**	**100**
	15895037^†^	2.11^e-20^	**96**	**99**
	15895042^*^	1.73^e-27^	**100**	**100**
	15895117^*^	1.01^e-24^	**100**	**94**
	15895150^*^	2.31^e-28^	**100**	**99**
	15895219	6.41^e-23^	**100**	89
	15895222	1.03^e-22^	**100**	89
	15895260^*^	8.42^e-29^	**100**	**100**
	15895327^†^	1.32^e-15^	83	**97**
	15895500^*^	1.02^e-27^	**100**	**99**
	15895509^†^	4.46^e-15^	84	**99**
	15895679^*^	2.12^e-26^	**100**	**99**
	15895776^*^	7.82^e-27^	**100**	**99**
	15895901^*^	2.31^e-28^	**100**	**100**
	15895970^*^	5.94^e-24^	**100**	**93**
	15895971^*^	2.79^e-27^	**100**	**99**
	15899571^*^	2.87^e-27^	**100**	**100**
	15899647	2.10^e-26^	**100**	**96**
Potri.T046100	250015	2.40^e-25^	**100**	**100**
(POPTR_0019s00240, *MET1*)	272305	9.58^e-26^	**99**	**100**
	272540	2.31^e-28^	**100**	**100**
	272567	2.87^e-27^	**100**	**100**
	275650	2.41^e-25^	**100**	**100**

Genes are annotated to the *P. trichocarpa* reference genome v3.0 (including v2.2 names and Arabidopsis homologs) and occur on five genes annotated to different chromosomes in v3.0 of the reference genome due to misassembly of the sex locus. The relative percent accuracy of SNPs in identifying females vs. males is based on the number of allelic variants matching sex-identified plants (through flowering observations). SNPs with >90% strict allelic distinction between sexes are highlighted in bold and are considered ‘strongly predictive’ for gender.

^†^SNP not reported in Geraldes *et al*.^15^. ^*^SNP reported associated with sex for *P. balsamifera* in Geraldes *et al*.^15^. ^a^GWAS analysis based on 126 trees. ^b^Includes 61 genotypes used for proof of concept and not included in the GWAS.

As a proof of concept, we directly sexed an additional 61 *P. trichocarpa* genotypes in the following year that were not included in our GWAS using field observations of flowering (Tables S1 and S2). We obtained SNP information for these 61 genotypes from whole genome sequencing, extracted the significant GWAS-identified SNPs, and sorted these markers based on flowering observations (male vs. female) to ascertain allelic frequencies by gender (Table 1). Together, the significant sex-associated SNPs clearly and correctly identified sexes of all 61 flowering *P. trichocarpa* genotypes that were not included in the GWAS (Table S2). Among these markers, 31 SNPs within four genes (*ARR17*, *GFG1*, *HEMA1*, *MET1*) were also considered ‘strongly predictive’ for gender, with <10% strict allelic variability differentiation between males and females (Fig. 2B, Tables 1 and S3). This refined suite of sex-associated SNPs within these four poplar genes (using v3.0 genome annotations) was then used to ascertain the genders of individuals with available genetic and phenotypic data but lacking flowering information.

Using these sex-predictive SNPs, we assessed 248 genotypes from the FLNRO collection and 548 *P. trichocarpa* genotypes from a collection by GreenWood Resources, OR (GW) and the BioEnergy Science Center, TN (BESC). In this second GW/BESC collection, genotypes originated from the southern-central portion of the species range (38.9–48.9°N, 121.7–123.7°W) (Table S2). We further extended our analysis to determine genders of the related species, *P. balsamifera* (following ref. 15). We assessed 473 genotypes from the Agriculture Canada Balsam Poplar (AgCanBaP) collection, with trees originating throughout the species range (46.1–68.6°N, 48.9–149.0°W), and matched field observations of flowering with sex-predictive SNPs from chromosomes 4, 9, 14 and 19 (using v3.0 genome annotations) (Tables S1 and S2). Combining field observations and sex-associated SNP markers from the SDR, we identified 469 female, 430 male and 5 putative sex-recombinant *P. trichocarpa* trees, and 219 female, 250 male and 4 putative sex-recombinant *P. balsamifera* trees (Table S2). Together, this sampling spans most of the geographical range for both species. Male and female genotypes showed relatively even numbers, indicating that the general sex ratio is likely 1:1 in both species, while the rarity of putative sex-recombinants indicates that the level of “SDR recombination” is very low (<1%).

### Sex-based phenotypic differences

We analyzed 96 non-reproductive traits for evidence of sexual dimorphism between males and females (excluding putative recombinants) in a wide range of features. A total of 70 functional traits influencing plant growth and survivability or how a plant gains or uses resources (including biomass, growth, phenology, physiology, leaf secondary compounds, disease resistance, drought stress and mortality) were tested between both genders. We also assessed 26 wood-related traits reflecting growth and carbon investment (ultrastructural anatomy and cell wall biochemistry) for evidence of sexual dimorphism.

Most traits were phenotyped at two plantation sites (Agassiz, BC, 49.25°N, 121.95°W; Totem Field, BC, 49.25°N, 123.10°W) in 858 sex-identified *P. trichocarpa* genotypes from the FLNRO and GW/BESC collections spanning juvenile to early reproductive phases (assessments of ~3000 individual trees across both sites, see Materials and Methods) (Tables 2 and S4). Traits were tested for sexual dimorphism with linear or mixed effects linear modeling (depending on whether genotypes were replicated within sites). We evaluated the significance of using ‘latitude’ as a covariate and proxy for the main axis of population structure in *P. trichocarpa* that occurs along the latitudinal species distribution^43, 46, 48^ (Fig. 1). We also tested for significant interactions between gender and latitude (cf. ref. 49). While we found that ‘latitude’ was a significant covariate for the vast majority of traits (and included this as a corrective factor in our models testing for sex-based differences, see Table 2), the interaction term was not significant for any trait. From these extensive datasets, we found little evidence for sexual dimorphism at any phase (i.e., from the establishment period to early reproductive stage) at either site (Tables 2 and S4). The few differences found between the sexes (based on *P*-values) were not robust as i) the results were not repeatable (within a season, from year-to-year and/or between sites), and ii) *P*-values would not be significant with the application of any multiple testing correction to reduce Type I error (Tables 2 and S4).

**Table 2:** Sex-based differences between male and female *Populus* individuals phenotyped for biomass, growth, phenology, disease, physiology, leaf anatomy and biochemistry traits across several years at different sites using linear or mixed effects modeling.

Trait	*P. trichocarpa* Totem Field, BC^a^		*P. trichocarpa* Agassiz, BC^b^		*P. balsamifera* Indian Head, SK^c^	
	Yrs	All trees (So. BC)	Yrs	All trees	Yrs	All trees
Biomass and growth						
Active growth rate^*^	2	NS^†^ (NS^§^)	1	NS	1	NS^†^
Basal diameter^‡^	3	NS^†^ (NS^§^)	3	NS^†^		
Bole density^*^	1	NS^†^ (NS^§^)				
Bole mass^*^	1	NS^†^ (NS^§^)			1	NS^†^
Bole volume^*‡^	5	NS^†^ (NS^§^)	3	NS^†^		
Bole volume gain^*^	3	NS^†^ (NS^§^)	2	NS^†^		
Bole volume growth rate (log value)	1	NS (NS)				
Branch angle	1	NS (NS^§^)				
Branch numbers^*^	2	NS^†^ (NS^§^)				
Diameter at breast height (DBH)	1	NS^†^ (NS^§^)				
Height^*‡^	4	NS^†^ (NS^§^)	3	NS^†^	1	NS
Height gain^*^	4	NS^†^ (NS^§^)	3	NS^†^	1	NS^†^
Height growth rate (log value)	1	NS^†^ (NS)			1	NS^†^
Height:diameter^*‡^	4	NS^†^ (0.023, NS^§^)	3	NS^†^		
Total woody biomass^*^	1	NS^†^ (NS^§^)				NS^†^
Disease and stress						
Establishment mortality	1	NS (NS)	1	NS		
Insect herbivory	1	NS (NS)				
*Melampsora* infection^*^	3	NS^†^ (NS^§^)				
*Septoria* infection					1	NS^†^
Spring drought stress	1	NS^†^ (NS)				
*Taphrina* infection	1	NS (NS^§^)	1	NS		
Physiology						
Carbon (per leaf area or mass)	2	NS (NS)			1	NS
Carbon (per wood mass)					1	NS
Carbon isotope ratio (leaf)^*^	2	NS^†^ (NS^§^)			1	NS^†^
Carbon isotope ratio (wood)	1	NS (NS^§^)			1	NS^†^
Carbon:nitrogen (C:N)^*^	2	NS^†^ (NS^§^)			1	NS^†^
Chlorophyll content – spring	1	NS (NS^§^)				
Chlorophyll content – summer^*^	2	NS^†^ (NS^§^)	1	NS^†^	1	NS^†^
Chlorophyll content – autumn	2	NS^†^ (NS^§^)				
Nitrogen (per leaf area or mass)^*^	2	NS (NS)			1	NS^†^
Nitrogen isotope ratio (leaf)^*^	2	NS^†^ (NS^§^)				
Nitrogen use efficiency (NUE)	2	NS^†^ (NS^§^)			1	NS^†^
Photosynthesis (area or mass-based)^*^	2	NS^†^ (NS^§^)			1	NS^†^
Stomatal conductance^*^	2	NS^†^ (NS)			1	NS
Water use efficiency (WUE)^*^	2	NS^†^ (NS^§^)			1	NS^†^
Leaf anatomy						
Canopy size					1	NS^†^
Leaf mass per area (LMA) – spring	2	NS (NS)				
Leaf mass per area (LMA) – summer^*^	3	NS (NS)	1	NS	2	NS^†^
Leaf mass per area (LMA) – autumn	2	NS (NS)				
Leaf shape	1	NS (NS^§^)	1	NS^†^	2	NS^†^
Leaf size			1	NS	2	NS^†^
Leaves per bud (terminal buds)^*^	2	0.049, NS^†^ (NS^§^)				
Lower surface stomatal density	2	NS^†^ (NS^§^)			1	NS^†^
Lower surface stomatal pore index	2	NS (0.042, NS)				
Upper surface stomatal density	2	NS^†^ (NS^§^)				
Upper surface stomatal pore index	2	NS^†^ (NS)				
Upper:lower stomatal density	2	NS^†^ (NS)				
Upper:lower stomatal pore length	2	NS^†^ (NS^§^)				
Leaf secondary compounds						
Phenolics – summer	3	NS^†^ (NS^§^)				
Phenolics – autumn	1	NS				
Tannins – summer	3	NS^†^ (NS^§^)				
Tannins – autumn	1	NS				
Phenology						
Bud break	3	NS (NS^§^)	2	NS^†^	1	NS^†^
Bud flush			2	NS^†^	1	NS^†^
Bud set^*^	3	NS^†^ (NS^§^)	2	NS^†^	1	NS^†^
Growth period^*^	2	NS^†^ (NS^§^)			1	NS^†^
Height growth cessation^*^	1	NS^†^ (NS^§^)				
Leaf drop^*^	3	0.015, NS^†^ (NS^§^)	1	NS^†^	1	NS^†^
Leaf flush	3	NS (NS^§^)	2	NS^†^	1	NS^†^
Leaf senescence (25%)	1	NS^†^ (NS^§^)	1	NS^†^	1	NS^†^
Leaf senescence (50%)	1	0.042, NS^†^ (NS^§^)	1	NS^†^	1	NS^†^
Leaf senescence (75%)^*^	1	0.026, NS^†^ (NS^§^)	1	NS^†^		
Leaf senescence (100%)^*^	1	NS^†^ (NS^§^)	1	NS^†^		
Maximum canopy period^*^	2	NS^†^ (NS^§^)			1	NS^†^
Maximum leaf lifespan	1	NS^†^ (NS^§^)				
Post-bud set period^*^	2	NS^†^ (NS^§^)	1	NS^†^		
Years to flowering	3	NS (NS^§^)				

*P*-values (<0.05) are reported here for all trees at each plantation and a subset of trees from southern British Columbia (BC). Full results, model selection and *P*-values listed in Tables S4, S5.

^†^Latitude and/or longitude are significant covariate(s) for tests of a given trait using linear or mixed effects modeling. ^§^Genetic population structure components (PC1 and/or PC2) are significant covariate(s) for tests of a given trait from the southern BC population using linear or mixed effects modeling. ^*^Q_*ST*_ values indicate the trait is adaptive (Keller *et al*. 2011, Porth *et al*. 2015). ^‡^Includes within-season monthly measurements. ^a^Males = 201, females = 235 (1972 trees total) assessed from the plantation at Totem Field, BC. For the southern BC subpopulation, males = 121 and females = 156 (1252 trees total). ^b^Males = 284, females = 300 (1010 trees total) assessed from the plantation at Agassiz, BC. ^c^Males = 92, females = 71 (no replication) assessed under greenhouse conditions at Indian Head, SK. For phenology and height traits, males = 238, females = 207 (2144 trees total) assessed from the plantation at Indian Head, SK.

Wood-related traits assessed for sexual dimorphism were phenotyped from *P. trichocarpa* samples collected at three plantation sites (Clatskanie, OR, 46.10°N, 123.20°W; Surrey, BC, 49.18°N, 122.85°W; Totem Field, BC, 49.25°N, 123.10°W) for 423 sex-identified genotypes from the FLNRO and GW/BESC collections spanning juvenile and sexually mature phases (Tables 3 and S4). Using the same statistical testing strategy for other traits (see above), we found that ‘latitude’ was largely not a significant covariate in our statistical models and that the interaction term with latitude was not significant for any trait. From these extensive datasets, we found no evidence for sex-based differences in any component of wood.

**Table 3:** Sex-based differences between male and female *Populus trichocarpa* trees in wood anatomy and cell wall composition traits phenotyped from trees of differing ages at three plantation sites using linear or mixed effects modeling.

Trait	9-year old	6-year old	4-year old
	Surrey, BC^a^	Clatskanie, OR^b^	Totem Field, BC^c^
	All trees (So. BC)	All trees	All trees (So. BC)
Wood composition and chemistry			
Alpha cellulose	NS (NS^§^)	NS	
Alpha cellulose:hemicellulose	NS (NS^§^)	NS	
Alpha cellulose:total lignin	NS^†^ (NS^§^)	NS	
Arabinose^*^	NS (NS)	NS	
C6:C5 sugars	NS (NS^§^)	NS	
Galactose^*^	NS^†^ (NS^§^)	NS	
Glucose	NS (NS^§^)	NS	
Glucose:xylose	NS (NS^§^)	NS	
Hemicellulose	NS (NS^§^)	NS	
Hemicellulose:total lignin	NS (NS)	NS	
Holocellulose	NS (NS)	NS	
Insoluble lignin	NS^†^ (NS^§^)	NS	NS (NS^§^)
Mannose	NS (NS^§^)	NS	
Rhamnose	NS (NS^§^)	NS	
Soluble lignin	NS (NS^§^)	NS	NS (NS)
Syringyl lignin monomers	NS^†^ (NS^§^)	NS	
Syringyl:guaiacyl lignin monomers	NS^†^ (NS^§^)	NS	NS^†^ (NS)
Syringyl monomers:soluble lignin	NS (NS^§^)	NS	
Syringyl monomers:total lignin	NS^†^ (NS^§^)	NS	
Total lignin	NS^†^ (NS^§^)	NS	
Wood cellulose crystallinity	NS (NS^§^)	NS	NS (NS^§^)
Xylose	NS (NS^§^)	NS	
Wood ultrastructural anatomy			
Coarseness			NS (NS)
Fiber length^*^	NS (NS)	NS	NS^†^ (0.014)
Microfibril angle (recent growth ring)^*^	NS^†^ (NS^§^)	NS	NS (NS^§^)
Wood density	NS^†^ (NS^§^)	NS^†^	NS^†^ (NS^§^)

*P*-values (<0.05) are reported here for all trees at each plantation and a subset of trees from southern British Columbia (BC). Full results, model selection and *P*-values listed in Table S4.

^†^Latitude is a significant covariate for tests of a given trait using linear or mixed effects modeling. ^§^Genetic population structure components (PC1 and/or PC2) are significant covariate(s) for tests of a given trait from the southern BC population using linear or mixed effects modeling. ^*^Q_*ST*_ values indicate the trait is adaptive (Porth *et al*. 2015). ^a^Males = 143, females = 189 (356 trees total) assessed from the plantation at Surrey, BC. For the southern BC subpopulation, males = 118, females = 154 (272 trees total). ^b^Males = 33, females = 30 (131 trees total) assessed from the plantation at Clatskanie, OR. ^c^Males = 156, females = 195 (773 trees total) assessed from the plantation at Totem Field, BC. For the southern BC subpopulation, males = 120, females = 155 (602 trees total).

All 96 phenotypic traits assessed in *P. trichocarpa* were re-analyzed for sexual dimorphism using a smaller subpopulation with limited isolation-by-distance to correct for local adaptation patterns (277 genotypes, 1252 trees; Fig. S2) and encompassed individuals originating in southern British Columbia^46^. All trees within this subpopulation were phenotyped at the Surrey and Totem Field plantations and were previously genotyped using a 34k SNP array^43^. We compared models using ‘latitude’ vs. two genome-wide SNP-based vectors (PC1 & PC2 from principal components analysis^43^) as corrections for population structure in our linear and mixed effects linear models. Results from the genetic-based models (i.e., using PC1 and/or PC2) were comparable to those using the geography-based models (Table S4), and we found that all traits with putative sexual dimorphism based on the larger population were not significant within this subpopulation (Table 2). Furthermore, we found limited evidence for sexual dimorphism (values in brackets in Tables 2 and 3). As with the other non-robust sex-based differences from analysis of the larger population, significance within this subpopulation was not reproducible across years (testing the same trees) and *P*-values were not small enough to survive multiple testing correction.

Finally, we repeated our analyses for evidence of sexual dimorphism using a set of 34 non-reproductive traits in *P. balsamifera*. Biomass, growth, phenology, physiology and leaf traits were phenotyped at Indian Head, SK (50.52°N, 103.68°W) in 445 sex-identified *P. balsamifera* genotypes (Tables 2 and S5). We considered both ‘latitude’ and ‘longitude’ as proxies for population structure following the continental species distribution and genetic structure of *P. balsamifera*^38, 50^. For most traits, at least one geographical variable was a significant covariate in our statistical models whereas geographical interactions with gender were not significant. From these extensive *P. balsamifera* datasets, we found no evidence for sex-based differences in any trait (Tables 2 and S5).

### Sex-based phenotypic variation across environments

To determine whether strong sex-based differences might occur depending on the environment, we used available data for the same genotypes cultivated at different plantation sites. Phenology and growth traits (bud break, bud set, and tree height) were recorded for 566 *P. trichocarpa* genotypes planted at three sites located within the species’ natural range (Agassiz = 49.25°N, Clatskanie = 46.10°N, Corvallis = 44.57°N). As with our previous assessments, ‘latitude’ was a significant covariate in statistical models for all traits tested at each site but the interaction term was not. There was no evidence for sex-based differences in the phenology traits assessed at any site (Table 4). At Clatskanie, a significant difference between males and females was obtained for tree height (*P* = 0.04), while this effect was not observed at the other plantations assessing the same set of genotypes. We determined the proportion of variance (ω^2^) attributable to sex for the traits measured at each site to assess the strength of sexual dimorphism, particularly regarding tree height at Clatskanie. We found that sex consistently accounted for a very low proportion of trait variance, including tree height at Clatskanie (<0.5%).

**Table 4:** Sex-based trait assessments between the same male and female *Populus trichocarpa* or *P. balsamifera* genotypes cultivated at plantation sites located across the species ranges.

Plantation site^a^	Yr	N	Bud flush^†^	Latitude	Sex	Bud set^†^	Latitude	Sex	Growth^†§^	Latitude	Sex
			(*P*-value)	(ω^2^)	(ω^2^)	(*P*-value)	(ω^2^)	(ω^2^)	(*P*-value)	(ω^2^)	(ω^2^)
*P. trichocarpa*											
Agassiz, BC (49.3°N)	2011	566	NA			0.63	0.31	0	0.28	0.11	0
Agassiz, BC (49.3°N)	2012	566	0.75	0.22	0	0.37	0.30	0	0.67	0.12	0
Agassiz, BC (49.3°N)	2013	566	0.94	0.17	0	NA			0.73	0.16	0
Clatskanie, OR (46.1°N)	2010	566	0.69	0.23	0	0.75	0.43	0	0.040	0.17	0.0047
Corvallis, OR (44.6°N)	2010	566	0.78	0.17	0	0.47	0.43	0	0.11	0.23	0.0021
*P. balsamifera*											
Fairbanks, AK (64.5°N)	2010	368	0.81	0.47	0	0.48	0.57	0	NA		
Indian Head, SK (50.5°N)	2010	368	0.81	0.23	0	0.39	0.79	0	NA		
Indian Head, SK (50.5°N)	2011	164	NA			NA			0.42	0.37	0
Prince Albert, SK (53.0°N)	2011	164	NA			NA			0.40	0.29	0

*P*-values report significance of likelihood ratio tests comparing linear models with and without sex included as a factor. Unbiased estimates of trait variance (ω^2^) attributed to either ‘latitude’ and ‘sex’ are included for all traits at each site.

^†^All linear and mixed effect models include geographical covariates (and planting year for the Agassiz plantation, see Methods). ^§^Tree growth evaluated as “height” in *P. trichocarpa* and “dry woody biomass” in *P. balsamifera*.

Using the same rationale, we also tested bud break and bud set in 368 *P. balsamifera* genotypes planted at two sites located near the northern and southern limits of the species range (Fairbanks (64.8°N, 147.7°W), Indian Head (50.52°N, 103.68°W)) and dry woody biomass measured for the same 164 *P. balsamifera* genotypes planted at two sites within the south-central portion of the species range (Indian Head (50.52°N, 103.68°W), Prince Albert (53.03°N, 105.78°W)). We found no effect of sex on any trait at any site, and the proportion of variance (ω^2^) attributable to sex was also negligible in *P. balsamifera* (Tables 4 and S5).

### Sex-based phenological response to spring warming

We tested whether inherent sex-based responses might exist depending on changing temperatures (as suggested by refs 3, 5 and 51), and specifically, whether spring phenology traits were altered by advancing spring temperatures through climate warming (e.g. ref. 52). We used a set of 422 sex-identified *P. trichocarpa* genotypes (genders determined within this study) to assess spring phenological response under changes to chilling time and higher temperature. In this controlled environment study, ‘early’ warming invoked a scenario of a shortened chilling/dormancy period followed by two different forcing temperatures (either 10 vs. 20 °C), while ‘later’ warming invoked a scenario with longer chilling (+68 days at 4 °C) followed by these same forcing temperatures. To compare sex-based responses in phenological timing within these scenarios, we used paired samples of genotypes under each temperature and recorded the time from planting dormant cuttings to occurrence of bud break and leaf flush (Table S6). Both chilling duration and forcing temperature had strong, interactive effects on the timing of bud break and leaf flush (Fig. S3; *P* < 0.0001); however, there was no evidence for interactions between gender and forcing temperature or chilling duration. Furthermore, timing differences in both bud break and leaf flush were not significantly affected by sex (*P* = 0.42, 0.38, respectively), indicating no gender effects on plant response to differing climate conditions.

## Discussion

Here, we present a case for ‘sexual homomorphism’ in plants, a term used for the absence of secondary (non-reproductive) sexual characteristics within a dioecious species^53^, alongside genomic evidence confirming a small SDR. We were able to examine gender-trait associations in a very great number of trees using reliable sex-associated SNP markers with extensive phenotyping to test sexual dimorphism in 96 non-reproductive traits, including many that directly influence plant fitness and performance. Strikingly, and despite the unprecedented size of the study, we did not find any reproducible trait association with gender, even within a subpopulation with less overarching population structure. While this result was against expectation, it suggests that apart from floral traits, gender is functionally and inherently neutral in both *P. trichocarpa* and *P. balsamifera*. This is all the more remarkable as sexual dimorphism in secondary sexual characteristics has been reported for many dioecious trees and shrubs, including poplars^5^. Finding equivalent patterns of trait variation between genders is unusual, but underscores that genetically-based sex does not necessarily result in inherent sexual dimorphism within a species. Together with the other negative report of sexual dimorphism in the distantly related *P. tremula*^33^, this might imply that sexual homomorphism is a rare characteristic of the genus *Populus*. We caution, however, that many previous studies identifying sexual dimorphism are often based on relatively small samples within species and/or do not control for relatedness within natural populations. Additionally, as local adaptive phenotypic differences can be strong (see below), even a small amount of population-based bias (or undetected clonality) in sampling might produce false positive results of sexual dimorphism.

### Implications for sexual conflict and genomic architecture in dioecious plants

Sexually antagonistic selection driven by sexual conflict is considered a main driver of the genomic architecture of sex chromosomes in dioecious taxa^11–14^. Under this sexual selection, sex-biased alleles becoming stably associated with the SDR expand the size and number of genes in the region causing selection for recombination suppression to spread. This episodic expansion of the non-recombining region produces genetic “strata” of different ages around the original sex locus and eventually may lead inexorably to fully differentiated, heteromorphic sex chromosomes^12–14^. Yet, the sex locus in *P. trichocarpa* has been shown to pre-date the evolutionary split between poplar species in *Populus* sections *Tacamahaca* and *Aigeiros*, suggesting that the SDR evolved ~7 Ma^15^. Notwithstanding this considerable estimated age, the SDR has remained highly compact. Results from the GWAS in this study using an expanded population were unable to extend the sex-associated region, confirming that the SDR is indeed small and lacks evidence for genetic “strata”.

One simple explanation for maintaining a small SDR in dioecious taxa is that sexual conflict is either low or suppressed over long periods of time, thereby removing one of the main drivers for sex chromosome expansion. This may not be peculiar to poplars, as other dioecious plant taxa have small SDRs (e.g., asparagus, grape, persimmon, strawberry)^12, 14^. In our study, the parallel finding of sexual homomorphism (i.e., no detectable significance of gender in non-reproductive traits), allows us to infer that selection through sexual conflict can be effectively unresolved and/or non-existent.

### Hypotheses on the absence of sexual conflict in dioecious plants

It is clear from this study that the evolution of separate males and females (primary sexual characteristics) can be uncoupled from the evolution of secondary sexual characteristics that promote differing fitness optima between both sexes. We propose two hypotheses for how this can potentially happen in dioecious species.

First, it is possible that negligible differences in reproductive resource investment are insufficient to support sexual conflict. Male and female plants can be expected to differ in the phenology and physiology of resource allocation (males invest in pollen pre-anthesis, whereas females invest, usually more heavily, in seed post-anthesis), and thus have different optima for physiological and phenological features^4, 7^. This normally leads to sexually antagonistic selection on genes underlying the traits in question, and in theory, resolving sexual conflict should lead to different phenotypes reflecting male and female fitness optima^4, 6–10^. Yet, this process may be absent in poplars and similar species because differential sexual resource investment is low compared to the greater investment in vegetative growth necessary for large trees and/or as a consequence of wind dispersal strategies^4^. Both genders have small (catkin) reproductive structures relative to large organismal stature, commonly observed in trees. In addition, wind pollination requires high levels of pollen in male plants, while wind fruit dispersal resulting in small seeds (with virtually no nutrient reserves) minimizes the usually greater reproductive investment by female plants.

Secondly, we suggest that sexual selection can be counteracted or minimized by swamping against a background of non-sex-related environmental selection, and propose that this may be true for other dioecious species with strong patterns of environmentally-based adaptation. Both *P. trichocarpa* and *P. balsamifera* have high levels of local adaptive differentiation^38, 41–43, 46, 54–56^, a common phenomenon in woody species in general^34^. They also have broad ranges that have been greatly affected by repeated glaciation patterns and quaternary climatic change (over the past 75 ka ref. 57). In both species, the current geographical distributions and adaptation patterns observed have been established within a more recent and much shorter time period compared with that of the sex locus (7 Ma^15^). This is particularly notable as numerous functional and fitness-related traits have complex genetic architectures^58^ and reflect strong selection for local optima^46, 55^.

Under this second scenario, it is therefore possible that the signal for sex-specific trait optima is much weaker and cannot survive overarching and swamping effects of strong, orthogonal selection from heterogeneous spatial and temporal environments. Alternatively, polymorphisms maintained by sexually antagonistic selection could be lost in the face of repeated adaptive sweeps for local conditions. If so, the interplay between sexual vs. environmental selection may be a critical factor to consider in the evolution of dioecy and sexual dimorphism in plants, a concept consistent with recent work showing an interaction between ecology and sexual selection in *Drosophila*^59^. Sexual conflict may effectively be stymied in dioecious species with strong adaptive selection, thereby inherently limiting the development and evolution of sex chromosomes and secondary sexual characteristics.

## Materials and Methods

### Plant materials

We used extensive collections of wild-sourced *Populus trichocarpa* Torr. & A. Gray and *P. balsamifera* L. (section *Tacamahaca*) genotypes for sex-based differences in non-reproductive traits between male and female trees. While considered “sister-species”, both have geographical ranges (and demographic histories) that are parapatric^57^. Data for all genotypes were obtained from individuals cultivated within common garden plantations (located within the natural range of the species). Most *P. trichocarpa* genotypes used in this study were originally collected by the British Columbia (BC) Ministry of Forests, Lands & Natural Resource Operations (FLNRO) across the northern two-thirds of the species range (44.0–59.6°N, 121.2–137.9°W) (Fig. 1A). This extensive collection was planted in 2000 at a nursery site in Surrey, BC (49.18°N, 122.85°W)^60^, and in 2008 at Totem Field, University of British Columbia, Vancouver, British Columbia (49.25°N, 123.10°W)^61^. Additional cuttings from the larger FLNRO collection were also planted in 2009 and 2010 at Agassiz, British Columbia (49.25°N, 121.95°W; this study). A second set of *P. trichocarpa* genotypes collected in California-Washington from the southern-central portion of the species range (38.9–48.9°N, 121.7–123.7°W) were obtained from GreenWood Resources (GW), Portland, OR and the BioEnergy Science Center (BESC), Department of Energy, Oak Ridge, TN. Cuttings from these GW/BESC genotypes were planted at Agassiz alongside the FLNRO collection and many of the same genotypes (from both the FLNRO and GW/BESC collections) were also planted in 2009 at two other field sites (Clatskanie, OR, 46.10°N, 123.20°W; Corvallis, OR, 44.57°N, 123.28°W)^42, 62^. Data from *P. balsamifera* were obtained from genotypes in the Agriculture Canada Balsam Poplar (AgCanBaP) collection made by Agriculture and Agri-Food Canada (AAFC), Agroforestry Division, Indian Head, Canada. Provenances of *P. balsamifera* originated from 65 populations throughout the species range throughout Alaska to New Brunswick (46.1–68.6°N, 48.9–149.0°W)^63^. Cuttings from this collection were established at three plantation sites (Indian Head in 2007, SK; 50.52°N, 103.68°W; Fairbanks in 2009, AK, 64.8°N, 147.7°W; Prince Albert in 2009, SK, 53.03°N, 105.78°W)^41, 56, 63^.

### Determination of sex-predictive SNPs

Tree sexes in *P. trichocarpa* were first established by field observations of male or female catkin development within the Surrey and Totem Field plantation sites during early spring months. Flowering was observed at Surrey in 2011–12 (when trees were 11–12 years old) and at Totem Field in 2012–15 (when trees were 4–7 years old). All genotypes observed flowering across years were consistently male or female and showed no evidence of sex lability.

A previous GWAS identified the sex locus and ca. 650 sex-linked SNPs based on 52 *P. trichocarpa* individuals (34 females, 18 males identified through field flowering observations) and 3.7 M SNPs from whole genome sequencing (WGS) mapped to version 2.2 of the reference genome^15^. We performed a similar analysis specifically to refine these results and focus on a smaller number of highly reliable “sex-predictive” SNPs annotated to the *P. trichocarpa* reference genome v3.0. We used 126 genotypes observed flowering in 2014 (67 females, 59 males) and employed GWAS with 2,248,597 bi-allelic SNPs from WGS and the binary sex phenotype (male vs. female). All individuals were previously fully sequenced and annotated to ‘Nisqually-1′ *P. trichocarpa* genome v3.0 (full details in ref. 47). SNPs were filtered for minor allele frequency of <0.05 and a genotyping rate of >0.9. The association analysis was performed using the ‘qtscore’ function implemented in the R package *GenABEL*^64^ and ran a logistic regression as follows:

1 $${\boldsymbol{y}}={\boldsymbol{\mu }}+X{\boldsymbol{\beta }}+{\boldsymbol{e}}$$

where **y** is a vector of binary observations (sex), ***μ*** is the intercept and overall population mean, **X** is the vector assigning fixed effects for genotypes, ***β*** is the fixed effects for SNP genotype, and ***e*** is the residual effect. We tested the association results with, and without, a population structure correction using principal components analysis (cf. ref. 58). Sex-associated SNPs were accepted after Bonferroni correction at α = 0.05 (significant at *P* < 2.26^e-8^).

### Gender identifications using sex-predictive SNPs

We extracted SNP information from 177 *P. trichocarpa* accessions (97 females, 80 males, including trees with field flowering observations in 2015 that were not used in the GWAS). We sorted significant SNPs into females vs. males and assessed each GWAS-identified SNP for its allelic form according to observed gender (i.e., proportion of allelic-specificity to either male or female). To be considered a reliable SNP marker for predicting sex in *P. trichocarpa*, we accepted only 10%, or less, difference between strict allelic variability and gender distinction. With this suite of predictive SNPs (i.e., loci with greater than 90% predictability for sex), we assigned tree sexes to *P. trichocarpa* genotypes in the FLNRO collection that had been sequenced^47^ but not previously been assessed for sex (either by field observations or genetics).

We also downloaded publicly available, independently sequenced SNPs annotated to the v3.0 of the genome for 548 *P. trichocarpa* genotypes from the Phytozome portal (http://phytozome.jgi.doe.gov/pz/portal.html). A total of 92 genotypes were sequenced independently by both data sources and allelic information for our suite of sex-predictive SNPs was compared across these individuals to check both data matching and reliability. We accepted a 5%, or less, mismatch between SNP calls made by both data sources (i.e., a limit of 3 out of 92 genotypes with non-identical SNP calls). From these assessments, we found ten predictive (i.e., clear allelic distinction between sexes) and consistent (i.e., same allele across both sequencing efforts) SNP markers on chromosomes 9, 14, 19 and scaffold 42 to assign tree genders to each genotype from the GW/BESC collection.

Finally, we applied our GWAS-identified SNPs on chromosomes 4, 9, 14 and 19 to assigning genders in *P. balsamifera* genotypes from the AgCanBaP collection. We extracted allelic information from 465 *P. balsamifera* accessions previously sequenced^47^, sorted individuals using 17 SNP markers identified in this study and found to be associated to sex in ref. 15, and compared our sex-identifications with field flowering observations for 84 accessions.

### Non-reproductive phenotypic trait assessments

A comprehensive set of 96 non-reproductive, functional traits (influencing plant growth and survivability or how a plant gains or uses resources) were obtained from detailed studies of *P. trichocarpa* trees grown in multiple common garden plantations (with replicated ramets). Many of the traits used in our study were previously described and data are available with their respective publications. Most sex identifications for *P. trichocarpa* and *P. balsamifera* individuals are from this study (see Results). At Totem Field, numerous phenology, biomass accumulation, growth, physiology and anatomical traits were studied from repeated measures of trees taken from the age of 1–5 years old (details in refs 43, 61 and 65). This same plantation was also monitored for severity of leaf rust (*Melampsora*) disease (details in ref. 66). We included additional information from Totem Field for establishment mortality, spring drought stress, leaf blister (*Taphrina*) disease, insect herbivory, and years to flowering (Appendix S1). At the Surrey site, increment wood cores were assessed from 9 year old trees determining wood anatomy and biochemistry (cell wall traits, fiber characteristics) (details in ref. 67). Increment wood cores of 6 year old trees from the GW/BESC collection grown at the Clatskanie site were also analyzed for these same wood traits (Klápště *et al*. unpublished). At Totem Field, wood trait data were assessed from basal discs of 4 year old trees harvested in 2012 (773 individuals) (Appendix S1). Phenotypic traits for these juvenile trees were measured following Porth *et al*.^67^. From these comprehensive datasets, we assessed trait differences for 436 sex-identified genotypes (with clonal replication = 1972 trees) at Totem Field, 332 sex-identified genotypes (with clonal replication = 356 trees) at Surrey, and 63 sex-identified genotypes (with clonal replication = 131 trees) at Clatskanie.

We also included assessments of traits obtained for all *P. trichocarpa* trees cultivated at the Agassiz plantation site (Appendix S1). Phenology and growth were recorded for all trees repeatedly from the age of 1–4 years following McKown *et al*.^43, 61^. Individual trees were also sampled once for numerous leaf traits corresponding to those taken at Totem Field (data from this study). From this dataset, we assessed trait differences for 584 sex-identified genotypes (with clonal replication = 1010 trees) at Agassiz. Similar phenotypic assessments (bud flush, bud set, tree height) were collected for the many of the same *P. trichocarpa* genotypes planted at the Clatskanie and Corvallis sites (details in ref. 42, see below).

We tested available phenotype data for *P. balsamifera*, including phenology, physiology, growth and leaf traits. At Indian Head, phenology, growth and leaf shape traits were measured in 445 sex-identified genotypes (with clonal replication = 2224 trees)^63^. We also assessed phenology traits recorded in the same year for 368 of these same genotypes planted at the Fairbanks site^56^. Canopy size, growth and physiology traits were obtained for 163 sex-identified genotypes from a large greenhouse study located at Indian Head^38^. Finally, we assessed dry woody biomass values for 164 sex-identified genotypes planted at both the Indian Head and Prince Albert sites and harvested after four years of growth (data from this study).

### Sex-based phenotypic trait analyses

We compared phenotypic differences between female and male trees using a step-wise approach. Traits that were measured across seasons, multiple years, and plantation sites were assessed independently due to unequal sample sizes and to determine whether any differences in tree sexes were attributable to plasticity (i.e., season, year of collection, tree age and/or plantation site). We used linear modeling (for traits without ramet replication) in the standard R package (R Core Team; http://www.R-project.org), or linear mixed effects modeling (for traits with ramet replication) in the *lme4* package^68^. For each trait, we first tested phenotypic-geographic covariation. We did not have adequate genetic information for all accessions tested to remove population structure using non-sex-linked SNPs (see below). In *P. trichocarpa*, we used ‘latitude’ as a proxy to remove most of the confounding variation as this correlates with the main axis of population structure^43^, encompasses demographic processes within the species^46, 48^, and as many traits have an adaptive component relating to a north-south geographical distribution^55^. In *P. balsamifera*, we used the same rationale and applied ‘latitude’ and ‘longitude’ as geographic covariates^50^.

Each trait was tested considering geographical variable(s) and ‘sex’ as fixed effects and for interactions between geography and gender. For traits measured at Agassiz, we included ‘planting year’ as a fixed effect on trait values (due to differing tree ages within the Agassiz plantation). All mixed effects models also included ‘genotype’ as a random effects term to account for replication due to multiple clonal ramets within a site. We used Bayesian Information Criterion (BIC) values to select a best-fit model on a trait-by-trait and test-by-test basis. For all traits analyzed, we found that interaction terms between geography and gender did not improve model fit and interaction terms were non-significant. *P*-values for all fixed effects were obtained using likelihood ratio tests (LRT) comparing a full model against the model with the fixed effect removed using the *lmerTest* package^69^. We reported sex differences with statistical significance at α < 0.05, but noted where these were non-repeatable and/or would fail to pass a multiple testing correction.

We used a similar approach in assessing traits between male and female *P. trichocarpa* genotypes from southern BC (genotypes shown to have less structure relating to local adaptation and isolation-by-distance, see Fig. S2; ref. 46) that were phenotyped at Totem Field and Surrey. Eigenvalues from principal components analysis based on 8 k non-sex-related SNPs were available for all southern BC accessions (details in ref. 43). We tested and compared trait models using ‘PC1’ and/or ‘PC2’ as genetic covariates and models with ‘latitude’ as a geographic covariate. We then applied the same statistical testing as described above for all phenotypic traits.

### Sex-based phenotypic trait variability across different environments

To test for potential sex-based differences across environments, we assessed three traits (bud flush, bud set, and tree height) measured in 566 *P. trichocarpa* genotypes originating from both the FLNRO and GW/BESC collection. All trees were cultivated in three plantations (Agassiz, Clatskanie, and Corvallis) located within the southern-central portion of the species natural range (44.57–49.25°N). Data from the Clatskanie and Corvallis plantations were collected within the same year (2010) and reported spatially-corrected genotypic means (not raw trait values) (details in ref. 42). For data collected at Agassiz, we used genotypic means (summarized by tree age) for traits collected throughout 2011–2013 (data from this study). We applied linear modeling using ‘latitude’ as a covariate and ‘sex’ as a fixed effect, adding ‘planting year’ as an additional fixed effect for Agassiz. Significance for fixed effects was assessed with LRT (see above). We calculated effect size using omega squared (ω^2^)^70^ as an unbiased representation of the proportion of total trait variance within the population due to either ‘latitude’ or ‘sex’ within the linear model.

We also tested three traits (bud flush, bud set, and woody biomass) measured in *P. balsamifera* genotypes common to three plantations (Fairbanks, Indian Head, and Prince Albert). Bud flush and bud set were measured within the same year (2010) for 368 genotypes at Fairbanks and Indian Head, while woody biomass was measured within the same year (2010) for 163 genotypes at Prince Albert and Indian Head. We applied the same linear modeling tests and calculations for omega squared (ω^2^) described above.

### Sex-based spring phenological response to warming temperatures

There is general interest in understanding how (and if) gender may play a role under climate warming^3, 5^, particularly with phenology (cf. ref. 52). We used a total of 422 *P. trichocarpa* genotypes originating from the FLNRO collection (see above) to test sex-based differences in the timings of both spring bud break and leaf flush under a controlled environment warming scenario (data and sex-identifications from this study, full details outlining the experimental framework available in Appendix S1). We used cuttings and randomly split genotypes into two groups with paired plantings under two scenarios: i) “early” warming (no additional chilling) testing sex-based responses at 10 °C vs. 20 °C (99 males, 110 females = 209 genotypes), and ii) “later” warming (with two months extended chilling) testing sex-based responses at 10 °C vs. 20 °C (96 males, 118 females = 214 genotypes). The first trial was planted after one month of chilling at 4 °C and the second trial was planted after three months of continuous chilling (i.e., an additional 68 days at 4 °C). Cuttings were monitored daily for appearance of leaves at the tip of the bud (indicating bud break) and emergence of leaves with petioles (indicating leaf flush). All data were first assessed for effects of population structure testing the significance of ‘latitude’ (see above). This covariate was not significant in any test. We then used mixed effects modeling considering ‘sex’, ‘scenario’ and ‘temperature’ as fixed effects, including interaction terms. For both traits, ‘scenario × temperature’ was significant. We then compared models using LRT to determine the contribution of ‘sex’ to plant response in each phenological trait.

## Electronic supplementary material

Supplemental Figs S1-S3

Supplemental Tables S1-S6
