# Author Correction: Sexual homomorphism in dioecious trees: extensive tests fail to detect sexual dimorphism in *Populus*

**DOI:** 10.1038/s41598-018-35443-y

**Published:** 2018-11-22

**Authors:** Athena D. McKown, Jaroslav Klápště, Robert D. Guy, Raju Y. Soolanayakanahally, Jonathan La Mantia, Ilga Porth, Oleksandr Skyba, Faride Unda, Carl J. Douglas, Yousry A. El-Kassaby, Richard C. Hamelin, Shawn D. Mansfield, Quentin C. B. Cronk

**Affiliations:** 1 Department of Forest and Conservation Sciences, Faculty of Forestry, University of British Columbia, Forest Sciences Centre, Vancouver, BC V6T 1Z4 Canada; 2 Department of Dendrology and Forest Tree Breeding, Faculty of Forestry and Wood Sciences, Czech University of Life Sciences, Prague, 165 21 Czech Republic; 3 Saskatoon Research and Development Centre, Agriculture and Agri-Food Canada, Saskatoon, SK S7N 0X2 Canada; 4 United States Department of Agriculture-Agricultural Research Service (USDA-ARS), Corn and Soybean Research, Wooster, OH 44691 USA; 5 Department of Wood Science, Faculty of Forestry, University of British Columbia, Forest Sciences Centre, Vancouver, BC V6T 1Z4 Canada; 6 Département des sciences du bois et de la forêt, Faculté de foresterie, de géographie et de géomatique, Université Laval, Québec, QC G1V 0A6 Canada; 7 Department of Botany, University of British Columbia, Vancouver, BC V6T 1Z4 Canada; 8 Present Address: Scion (New Zealand Forest Research Institute Ltd.), Whakarewarewa, Rotorua 3046 New Zealand

Correction to: *Scientific Reports* 10.1038/s41598-017-01893-z, published online 12 May 2017

The original version of this Article contained an error in the title of the paper, where “Sexual homomorphism in dioecious trees: extensive tests fail to detect sexual dimorphism in *Populus*” was incorrectly given as “Sexual homomorphism in dioecious trees: extensive tests fail to detect sexual dimorphism in *Populus*^†^”. This has now been corrected in the PDF and HTML versions of the Article.

