# Supplementary material for: Sexual homomorphism in dioecious trees: extensive tests fail to detect sexual dimorphism in Populus
Source: Sci Rep. 2017 May 12;7:1831. doi: 10.1038/s41598-017-01893-z (PMC5431824; doi:10.1038/s41598-017-01893-z)
Supplement: Supplementary file 1 — Supplemental Figs S1-S3 [file 41598_2017_1893_MOESM1_ESM.pdf]

Supplementary Figures in support of "Sexual homomorphism in dioecious trees: extensive tests fail to detect sexual dimorphism in *Populus*"

Authors: Athena D. McKown, Jaroslav Klápště, Robert D. Guy, Raju Y. Soolanayakanahally, Jonathan La Mantia, Ilga Porth, Oleksandr Skyba, Faride Unda, Carl J. Douglas, Yousry A. El-Kassaby, Richard C. Hamelin, Shawn D. Mansfield, Quentin C. B. Cronk

List of Supplemental Figures

Fig. S1. Q-Q and Manhattan plots from two genome-wide association studies (GWAS) for loci associated with sex in *Populus trichocarpa* using 126 sex-identified individuals.

Fig. S2. Spread of 277 genotypes from southern British Columbia with high gene flow and limited isolation-by-distance.

Fig. S3. Sex-based spring phenological responses (genotypic mean value ± SD) in male and female *Populus trichocarpa* genotypes grown under controlled treatments manipulating temperature and duration of chilling.

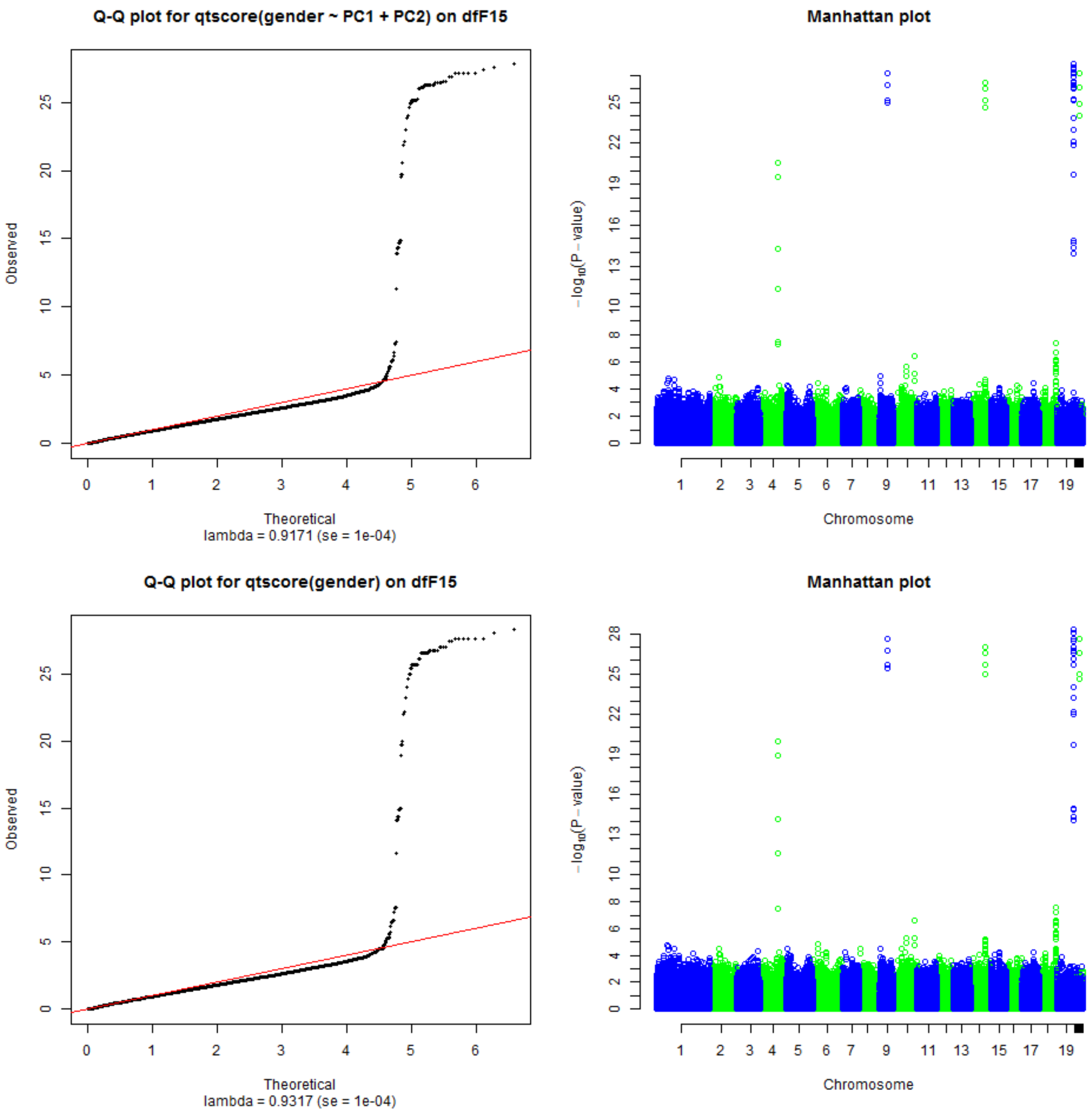

Fig. S1. Q-Q and Manhattan plots from two genome-wide association studies (GWAS) for loci associated with sex in *Populus trichocarpa* using 126 sex-identified individuals. Top right and left panels show association results using a population structure correction (PC1 & PC2 developed using principal components analysis). Bottom right and left panels show association results using a simple model (no structure correction implicated). Both analyses yield the same results and model fit (based on lambda scores noted on the Q-Q plots) is equivalent.

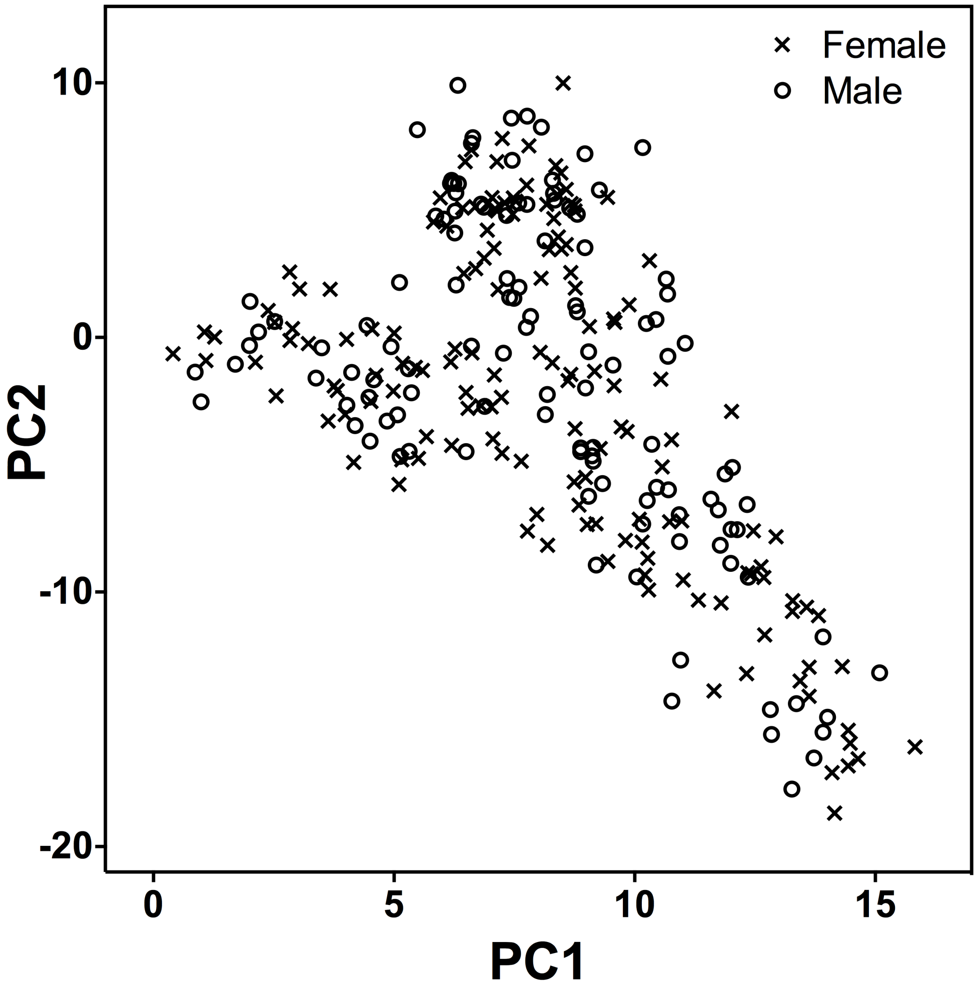

Fig. S2. Spread of 277 genotypes from southern British Columbia with high gene flow and limited isolation-by-distance along two principal components (PC1&PC2) developed through principal component analysis of 8k SNPs (SNP data and principal components from McKown et al. 2014a, identification of southern British Columbia subpopulation from Geraldes et al. 2014).

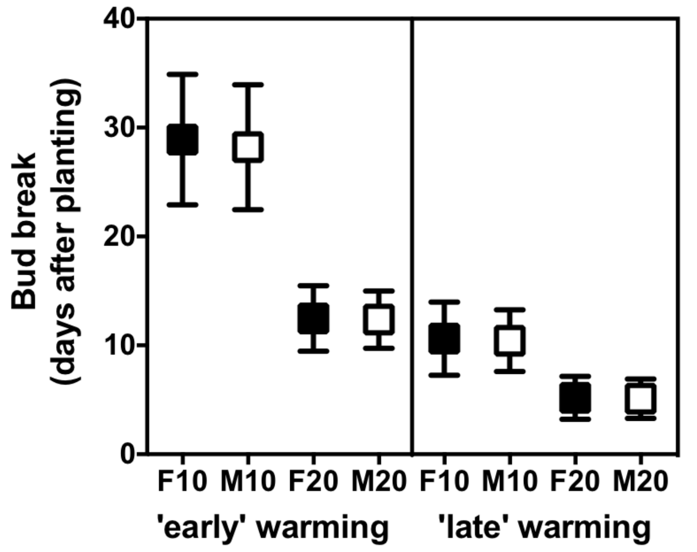

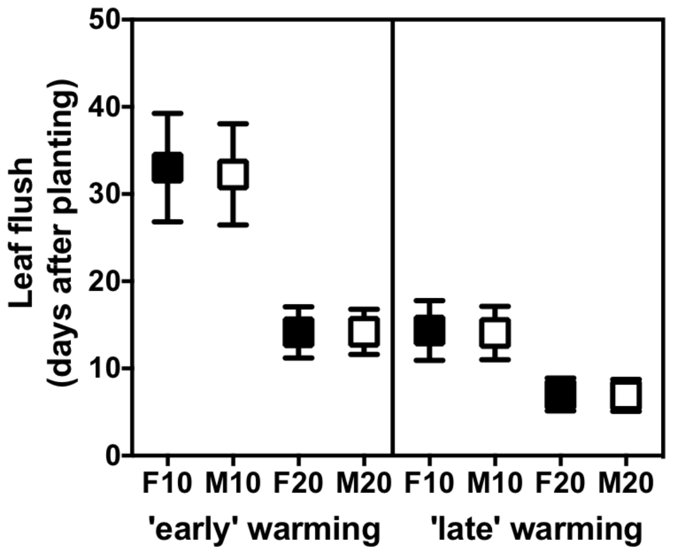

Fig. S3. Sex-based spring phenological responses (genotypic mean value ± SD) in male and female *Populus trichocarpa* genotypes grown under controlled treatments manipulating temperature and duration of chilling. 'Early' warming (no additional chilling) and 'later' warming (with two months extended chilling) are both followed by forcing temperatures of 10 °C vs. 20 °C. No significant differences attributable to sex were found for either bud break or leaf flush using mixed effects modeling (black=female, white = male) (full results and *P*-values listed in Table S7).
